# Native Non-*Saccharomyces* Yeasts as a Tool to Produce Distinctive and Diverse Tamjanika Grape Wines

**DOI:** 10.3390/foods11131935

**Published:** 2022-06-29

**Authors:** Ivana Karabegović, Marko Malićanin, Nikola Popović, Sandra Stamenković Stojanović, Miodrag Lazić, Jelena Stanojević, Bojana Danilović

**Affiliations:** 1 Faculty of Technology, University of Niš, Bulevar Oslobodjenja 124, 16000 Leskovac, Serbia; sandrastamenkovic@tf.ni.ac.rs (S.S.S.); lazic@tf.ni.ac.rs (M.L.); jstanojevic@tf.ni.ac.rs (J.S.); bojana.danilovic@junis.ni.ac.rs (B.D.); 2 Faculty of Agriculture, University of Niš, Kosančićeva 4, 37000 Kruševac, Serbia; malicanin.marko@ni.ac.rs; 3 Institute of Molecular Genetics and Genetic Engineering, University of Belgrade, Vojvode Stepe 444a, 11042 Belgrade, Serbia; popovicnikola@imgge.bg.ac.rs

**Keywords:** indigenous yeast isolates, non-*Saccharomyces* yeasts, volatile compounds, sensory evaluation, Tamjanika

## Abstract

The enological potential of two previously characterized indigenous yeast isolates, *Hanseniaspora uvarum* S-2 and *Candida famata* WB-1, in pure and sequential inoculation with commercial yeast *Saccharomyces cerevisiae* QA23 were analyzed in industrial-scale vinification of the grape variety Tamjanika. Their contribution to the quality and aroma profile was investigated by quantifying volatile compounds and wine sensory evaluation. Both yeast isolates were able to complete alcoholic fermentation, to reduce ethanol concentration up to 1.06% *v*/*v* (in monoculture) in comparation to *S. cerevisiae* QA23, and to enhance aroma and sensory profile. Based on calculated odor activity values (OAV), p-cymene, ethyl hexanoate, ethyl octanoate, and ethyl decanoate were the major aroma volatile compounds in all Tamjanika wine samples. Analyzed yeast strains significantly affected relative contribution of volatile compounds and can be considered responsible for the differences and uniqueness of the obtained wine samples. Besides confirmation of good enological and fermentative characteristics, selected isolates can be characterized as high ester-producing strains with potential to enhance the floral and fruity aromas of wine. The present study represents a further step toward the use of indigenous yeast isolates at industrial-scale fermentation in order to ensure the regional signature of Tamjanika wine.

## 1. Introduction

In recent years, many studies have been unravelling the enological potential of non-*Saccharomyces* yeasts and their impact on the quality and aroma complexity of the wine. Yeasts are widespread in nature, commonly occurring on sugar-rich substrates such as fruits, but also in soil, plant, and in different natural ecosystems [1,2]. Although grape variety, vineyard practices, and winemaking protocol highly contribute to the wine quality, the fact that the majority of compounds responsible for the wine sensory and quality characteristics are formed during the fermentation indicates that the wine microbiota can be considered crucial for the wine distinctiveness and authenticity [3]. Along with *Saccharomyces* yeasts, which play a predominant role as the main fermentation species, several non-*Saccharomyces* species have attracted considerable attention due to their good enological properties. Recent studies have shown that their winemaking potential relies on the ability to produce wines with lower ethanol content or volatile acidity, release the varietal aroma from non-volatile precursors, enhance wine complexity, and prevent spoilage [3,4,5,6,7]. *Candida* and *Hanseniaspora* are the main yeast genera naturally present in the early stages of alcoholic fermentation (up to 5–6% *v*/*v* ethanol content), while, subsequently, *Saccharomyces cerevisiae* becomes predominant and completes the fermentation process. Despite the fact that indigenous non-*Saccharomyces* yeasts are represented only in the first stage of alcoholic fermentation, they possess the ability to produce high levels of aromatic compounds such as esters, higher alcohols, and fatty acids, sufficient to modify the volatile profile and sensory quality of wines [8]. In proper inoculation protocols, non-*Saccharomyces* yeasts in sequential or simultaneous inoculation with *Saccharomyces* yeasts could significantly contribute to the wine quality while mitigating the risks of stuck/sluggish or spoiled fermentations [4,6,9].

Knowing that the representatives of *Hanseniaspora* and *Candida* genera mainly initiate the fermentation and remain dominant during the initial stage [10,11,12], these strains can be considered as possible candidates for the non-*Saccharomyces* starter cultures. Furthermore, the use of indigenous yeast strains as starter cultures could additionally contribute to the unique qualities of wine, enhancing distinctiveness and regional characteristics of the wine. Previous reports indicated that although *Hanseniaspora* yeast demonstrates low fermentative power, it has a positive contribution on the wine quality related to the high production of primary (glycerol, acetaldehyde) and secondary (higher alcohols, acetate and ethyl esters, medium-chain fatty acids) metabolites [12,13,14]. Yeasts from *Candida* genera are characterized as high glycerol and terpenol producers, and described as lower producers of aldehydes, acetic acid, and acetate esters [4]. Although *Hanseniaspora* spp. are known as producers of undesirable compounds (acetic acid, sulfur compounds, etc.), a drastic strain variability was observed so some strains might, therefore, produce these secondary products in an acceptable or lower level [12,15]. In addition, *Hanseniaspora* spp. and *Candida* spp. have the ability to excrete a broad range of extracellular enzymes which have an irreplaceable role in releasing aroma compounds from non-volatile precursors [12,15,16], promoting the extraction of pigments from the grape skins, hydrolyzing proteins and promoting cell autolysis [4]. Further, few authors noted that some *Hanseniaspora* or *Candida* strains in mixed culture with *S. cerevisiae* could enhance the mouthfeel, flavor, and quality of wines [10,13,15,17].

A hunting campaign for non-exploited yeast strains with good enological potential mainly focuses attention on the microbial communities naturally associated with vineyard or cellar. In order to find tailored starter cultures for producing unique aroma profiles and give regional signatures to the wines, it should be extended further to the surrounding ecosystems. Additionally, the majority of the published papers deal with the enological characterization of the non-*Saccharomyces* yeasts such as metabolism/enzymatic details or winemaking potential mainly in the laboratory-scale level, lacking information about scale-up procedures and their effect on commercial fermentations. Factors such as geometry and volume capacity, heterogeneity in nutrient or oxygen distribution, or rapid sedimentation of yeast cells may be responsible for differences arising in scale-up procedures [12,18].

In our previous research, an extensive number of non-*Saccharomyces* yeasts belonging to epiphytic microbiota of regionally grown fruit were enologically characterized in order to find non-exploited yeast strains with good enological potential [14,19,20]. The novelty of the present study is directed in the improvement of the insufficient knowledge about the specific impact of selected yeast strains in the industrial level fermentations in pure and mixed culture with *S. cerevisiae*. In this study, the contribution of the selected native *Hanseniaspora uvarum* S-2 and *Candida famata* WB-1 strains was assessed by analyzing the kinetics of alcoholic fermentation, standard enological parameters, and volatile and sensorial profile of wines produced from the grape cultivar Tamjanika (local name of the international Muscat Blanc variety). Despite the fact that this aromatic white grape variety has been very popular in Serbia for the production of wines with pronounced fruit and floral aroma, there is limited knowledge available about the chemical and volatile profiles of Tamjanika wines produced in Serbia. Hence, the significance of this research is also to provide an insight into the aroma and quality characteristics of the Tamjanika wines.

## 2. Materials and Methods

### 2.1. Yeast Strains

The *H. uvarum* S-2 and *C. famata* WB-1 were previously isolated from the epiphytic microbiota of regionally grown fruit, characterized for their enological properties, and maintained at −20 °C in glycerol [14,19]. Isolates were identified by API 20 C AUX system (bioMérieux, Marcy l’Etoile, France) and results were confirmed by PCR analysis according to ITS sequence (data not shown). Prior to the fermentation, the yeast inoculum of both strains was separately prepared in a 2.5 L laboratory glass bioreactor with mechanical stirring (KLFM Bioengineering, Wald, Switzerland) under previously optimized growth conditions (data not shown). After the cultivation, strains were centrifuged (Th16B centrifuge, Zhengzhou, China), washed twice with sterile distilled water, and suspended in grape juice. For sequential and control fermentation, commercial strain *Saccharomyces cerevisiae* QA23 (Lallemand, Montreal, QC, Canada) was used according to the producer’s instructions.

### 2.2. Wine Production

Tamjanika grapes were cultivated in the Trstenik wine-growing subregion, Central Serbia (43°33′ N, 21°03′ E, continental climate, single Guyot vine training system) and processed at the “Marco” winery (Bučje, Serbia). Must characteristics were 23.6 Brix, 5.95 g/L total acidities, and pH 3.4. Manually harvested grapes were destemmed and crushed. After the addition of potassium-metabisulfite (6 g/hL), pectolytic enzyme (Lallzyme Cuvée Blanc, Lallemand, Montreal, QC, Canada, 3 g/hL), and vitamin C (5 g/hL), before further pressing, the grape juice remained in contact with the grape skin and seeds, for 24 h at a temperature of 4 °C. The free-run must was clarified (static sedimentation 24 h at 8–10 °C) and then divided into five stainless-steel fermentation tanks (1200 L). Fermentations were performed in duplicate. Inoculation of must for pure and sequential fermentation was performed with prepared culture of native strains *H. uvarum* S-2 or *C. famata* WB-1 with the final cell number of 1 × 10^6^ CFU/mL. For sequential fermentation protocol, after the initial fermentation stages, when °Brix decreased by 3 degrees, inoculation with *S. cerevisiae* QA23 (25 g/hL) was performed. The control wine sample was inoculated only with *S. cerevisiae* QA23 (25 g/hL). Yeast nutrient Fermaid E (Lallemand, Montreal, QC, Canada) was added in the concentration of 40 g/hL. All wines were fermented to dryness. The kinetic of alcoholic fermentations was monitored by a decrease in sugar concentration (digital refractometer DR6000, Krüss Optronic GmbH, Hamburg, Germany). At the end of alcoholic fermentation. Wines were sulphited (25 g/hL). After two months, wines were finned (bentonite, 1 g/L), filtered by Seitz filter plates K 100 (Pall Seitz, Drieich, Germany), and bottled.

### 2.3. Standard Physicochemical Analyses

The alcoholic strength, total dry extract, total and volatile acidity, pH, reducing sugar, and free and total SO_2_ were determined according to the methods proposed by the International Organization of Vine and Wine (OIV, 2021). All measurements were performed in triplicate and results were expressed as the mean value with standard deviation.

### 2.4. Determination of Wine Volatile Compounds Composition (HS-SPME-GC-MS)

The volatile aroma compounds were determined by solid-phase microextraction coupled with gas chromatography, following the procedure described in detail in our previous papers [20,21,22]. The SPME fiber Divinylbenzene/Polydimethylsiloxane//Carbon Wide Range/Polydimethylsiloxane (DVB/PDMS//Carbon WR/PDMS; 50/30 µm thickness, Supelco, Bellefonte, PA, USA) was used for the extraction of volatiles. Concentrations of volatile compounds (expressed in mg/L) in the wine samples were determined by an external standard method and expressed as mean of two injections of each replicate.

### 2.5. Sensory Analysis

The sensory profile of the Tamjanika wine samples was evaluated (ISO 6658, 2017; ISO 3591, 1977; OIV, 2015) by nine judges officially certified for wine sensory analysis by the Serbian Ministry of Agriculture, Forestry and Water Management (six females and three males aged from 31 to 53 years old). Most significant attributes were defined for Tamjanika wines by the assessors on consensus, while a short training session with reference standards was provided monthly for all the assessors to avoid any bias during the sensory evaluation. A ten-point intensity scale (1 = “extremely low”, 5 = “moderate”, 10 = “extremely high”) was used to rate the olfactory (spice, tobacco, herbal, citrus, tropical fruit, dry fruit, fresh fruit, floral, toasted, complexity, intensity, and typicality) and gustatory (harmony, acidity, astringency, fullness, complexity, duration, intensity, and typicality) attributes. The samples were randomly numbered and presented to the panelists. Tastings were conducted at room temperature, while unsalted crackers and room-temperature water were provided for mouth rinsing between each sample. The analysis was performed in duplicate and the results are expressed as mean values in a spider chart (Microsoft Excel, version 2016, Microsoft Corporation, Redmond, WA, USA).

### 2.6. Statistical Analysis

Statistical analyses of the data were performed using the SPSS^®^ 26.0 (IBM^®^ SPSS Statistics Software, New York, NY, USA, trial version) and STATISTICA 7 (StatSoft Inc., Tulsa, OK, USA., trial version) software. Shapiro–Wilk and Levene’s tests were performed to evaluate the normality of data distribution and homogeneity of variances, respectively. Statistical differences were determined by one-way ANOVA followed by Tukey’s HSD test, while Kruskal–Wallis test with post hoc Dunn’s test were applied for variables that did not meet normality criteria. A significance level of 0.05 was used for all statistical analyses. Principal component analysis (PCA) was performed for the differentiation of samples based on the aroma profile of wines.

## 3. Results and Discussion

### 3.1. Fermentation Kinetics

Non-*Saccharomyces* yeasts in pure or sequential fermentation with *S. cerevisiae* QA23 were used as an alternative solution to overcome the drawback of a single commercial species process and to improve the complexity, sensory properties, and flavor of wines [10,12,23]. Additionally, more attention is paid to non-conventional indigenous yeasts with good enological potential, with the aim to differentiate wine productions and ultimately shape and contribute to the regional wine characteristics [24,25,26,27,28,29].

**Figure 1:**
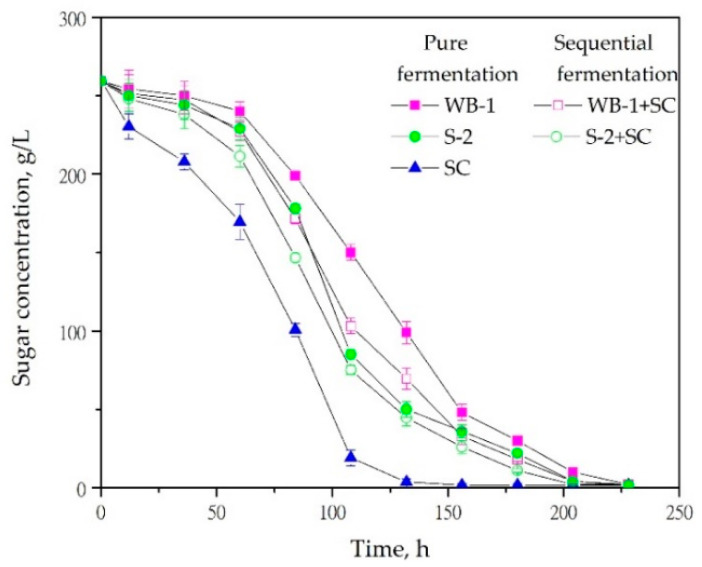
Kinetics of pure and sequential fermentations inoculated with two non-*Saccharomyces* isolates (*C. famata* WB-1 and *H. uvarum* S-2) and *S. cerevisiae* QA23 (SC). Data were the mean ± SD of two biological replicates of fermentation.

In our previous studies, we showed that some commercial and native non-*Saccharomyces* yeasts have a positive impact on the quality, volatile, and sensory profile of Prokupac red wines [14,19,21]. Since the results were demonstrated at laboratory level, the logical continuation of the research was validation at the industrial level. The first step was examination of sugar consumption kinetics in pure and sequential fermentations (Figure 1).

Regardless of the yeast strain or fermentation protocol, a typical fermentation curve was observed for all fermentation trials. In the control sample, fermentation was completed 5 days after inoculation, while for the non-*Saccharomyces* strains, fermentation lasted for more than 8 days. About half of the sugar amount in grape juice was depleted after 70 h of fermentation by *S. cerevisiae* QA23, while more than 80% of sugar remained not consumed by *C. famata* WB-1 and *H. uvarum* S-2 strains, regardless of the fermentation protocol, even after 90 h of inoculation. Among both used non-*Saccharomyces* yeasts, faster reduction of sugar content was observed for *H. uvarum* S-2, independently on the fermentation protocol, indicating slightly better fermentation ability of this strain. Furthermore, after co-inoculation with commercial yeast *S. cerevisiae* QA23, the fermentation was accelerated compared to the pure fermentations with *C. famata* WB-1 and *H. uvarum* S-2. However, both pure fermentations required an additional 24 h for dryness to be attained, compared to the sequential fermentation inoculated with the same non-*Saccharomyces* isolates. Additionally, during the monitoring period, all fermentations were performed with no stagnation. Good fermentation ability of *C. famata* WB-1 and *H. uvarum* S-2 was consistent with our previously published results for the laboratory fermentation trials [14,19]. Similar fermentation abilities were earlier reported for *H. uvarum* strains isolated from spontaneous fermentation of Negroamaro [13] or Montepulciano d’Abruzzo and Trebbiano [30] grapes and for some other non-*Saccharomyces* yeasts such as *Candida zemplinina* [30], *Lachancea thermotolerans* [31], *Torulaspora delbrueckii*, *Kluyveromyces thermotolerans* [32], and several species of the genus *Kazachstania* [33]. Such fermentation characteristics indicated that both tested non-*Saccharomyces* yeasts (*C. famata* WB-1 and *H. uvarum* S-2) could be considered as possible starter cultures in industrial-scale vinification, without the risk of stuck or sluggish fermentations. Moreover, the slower alcoholic fermentation observed for tested isolates does not necessarily represent a disadvantage, especially when bearing in mind that slower fermentation rate results in a cooler fermentation, leading to better retention of volatile compounds and lower demand for energy, which prevents overheating during the fermentation [34].

### 3.2. Standard Quality Parameter of Wine

Basic quality parameters for produced wines clearly indicated that yeast strain and fermentation protocol significantly affect the Tamjanika grape wine characteristics (Table 1). Compared to the control wine sample, the fermentation with native yeast strains, in both fermentation protocols, significantly reduced the concentration of ethanol. Precisely, the concentration of ethanol was lower in the samples produced in sequential fermentations compared to the control, from 0.36% *v*/*v* to 0.53% *v*/*v*, for the *H. uvarum* S-2 and *C. famata* WB-1, respectively, while the highest ethanol reduction (1.06% *v*/*v* less compared to the control) was found in the wine sample produced with monoculture of *C. famata* WB-1 strain. Such results indicated that selected strains can be used to reduce the content of ethanol in large-scale wine production, which is in line with the interests of modern winemaking and consumer demands for products with a lower content of ethanol. The ethanol reduction was earlier confirmed for non-*Saccharomyces* species, and this ability highly depends on the yeast strain or/and fermentation conditions [35,36,37]. Although statistically significant differences in residual sugar content are observed between produced wines, all the fermentations reached dryness (reducing sugar lower than 4 g/L). Lower ethanol concentration and similar sugar consumption for used yeast strains compared to the commercial *S. cerevisiae* QA23 could be explained by the ability of certain yeast strains to produce different metabolic byproducts (glycerol, succinic acid, lactic acid) or to accumulate greater yeast biomass [38].

Although in our previous papers we found that *C. famata* WB-1 and *H. uvarum* S-2 isolates were not able to ferment sugar to dryness in the sterile must, we highlighted that both isolates can produce dry wines from fresh must in a non-sterile environment that is more similar to the real conditions during wine fermentation. Completed fermentation of fresh Prokupac grape, or Tamjanika grape must in this work, confirmed the presence of grape or winery indigenous microbiota, which is able to prevail and finish the fermentation. It is worth mentioning that in our previous experiment we found that the inoculation with 6 log CFU/mL should be considered sufficient to ensure the growth and proliferation of inoculated isolates and their predominance at least during the early stages of fermentation, which will result in a substantial impact on aroma and sensory quality of produced wines. It has been reported that although non-*Saccharomyces* yeasts are mainly present in the first stage of fermentation, they significantly affect the characteristics of the wine [39]. This is not just based on the direct production of secondary metabolites, but related to the enzyme production responsible for the bioconversion of nonvolatile and odorless flavor precursors into their active version [40]. The total and volatile acidity of wines produced by different yeast isolates or fermentation protocols reveal slight differences. A higher level of total acidity was found in the wines produced by non-*Saccharomyces* yeast strains in monoculture. These findings are in line with our previous results for *C. famata* WB-1 strain [19]. This ability should be considered a positive characteristic, especially in warm climate areas where the trend of the total acidity reduction in wines is observed [41,42]. Independently of the fermentation protocol, volatile acids were slightly higher in wines produced with *C. famata* WB-1 and *H. uvarum* S-2, but still, in all produced wines, this level was far below the limits proposed by the OIV (1.2 g/L of acetic acid). Although different non-*Saccharomyces* yeasts (such as *Candida krusei*, *Candida stellate*, *Hansaniaspora uvarum/Kloeckera apiculate*, *Pichia anomala, Saccharomycodes ludwigii*) are known as the producers of higher amount of acetic acid [43], this characteristic also depends on sugar concentration, nitrogen source, pH, and must composition [44]. Comi and co-workers [45] reported that from forty-nine apiculate strains, just a few produced acetic acid above 1 g/L. It is important to take into consideration that yeast strains behaved differently in different musts, which could be explained by the fact that fermentation medium provides a very selective environment [32]. Compared to our previous results [14,19,20], the effect of the same native yeasts strains (*C. famata* WB-1, *H. uvarum* S-2) on wine total acidity were different for Prokupac and Chardonnay grape must. Such results emphasize the need to examine the enological potential of different indigenous yeast for each specific grape variety individually in order to find the perfect yeast–grape variety match. Compared to the wines produced with native yeast isolates in both fermentation protocols, the lowest total extract content was present in the control wine sample. Significantly higher content of total dry extract (up to 15 and 32% in sequential and pure fermentation protocol, respectively) was present in the wine produced with *H. uvarum* S-2 compared to the control sample, which is in accordance with the literature data [19] and could be explained by the ability of *H. uvarum* strains to produce high content of glycerol or other non-volatile compounds [21]. Bearing in mind that wines with a total extract content below 20 g/L are characterized as thin and light-bodied [20], such results indicate that the application of *C. famata* WB-1 or *H. uvarum* S-2 could positively contribute to the body and mouth-feel of Tamjanika wines.

### 3.3. Sensory Evaluation

**Figure 2:**
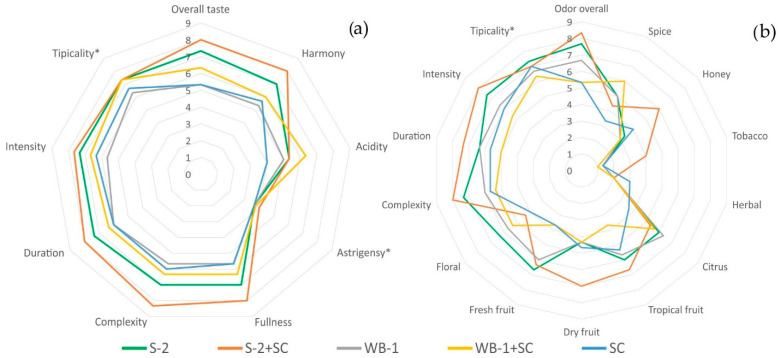
Gustatory (**a**) and olfactory (**b**) attribute scores for Tamjanika wine samples produced in pure and sequential fermentations inoculated with a two non-*Saccharomyces* strains (*C. famata* WB-1 and *H. uvarum* S-2) and *S. cerevisiae* QA23 (SC). Data were the mean of two biological replicates of fermentation. Asterisks indicate that there is no significant differences between the means in attribute intensities (*p* < 0.05, Tukey’s HSD test).

To evaluate the impact of two native non-*Saccharomyces* yeast strains (*C. famata* WB-1, *H. uvarum* S-2) in pure or sequential fermentation on Tamjanika wine sensory profile, a sensory evaluation was carried out (Figure 2). Slight differences in the gustatory (taste) attribute scores of the wines produced by different yeast strains and fermentation protocols were found, while more pronounced differences were observed for olfactory attribute scores. Still, significant variation in taste and odor scores was detected among all wine samples (*p* < 0.05), except for the taste typicality and astringency (*p* > 0.05).

Generally, Tamjanika wines produced in all fermentations resulted in similar taste profile for the wines produced with the same non-*Saccharomyces* yeast, but with statistically different scores for the majority of gustatory attributes. The increase in fresh fruit, citrus, and floral odor attributes was observed in wines produced in fermentation with pure *C. famata* WB-1 and *H. uvarum* S-2 compared to the control or wine produced in sequential fermentation. The wine produced in sequential fermentation with *H. uvarum* S-2 was highly rated for all taste attributes, except for the acidity, achieving the highest score for the honey, tropical, or dry fruit olfactory attribute. Additionally, this wine sample had the best scores for complexity (8.3), duration (8), fullness (8), intensity (7.7), and harmony (8). Samples fermented by *C. famata* WB-1 in sequential fermentation were characterized by the highest score for acidity and spice aroma notes. Compared with the control sample, used non-*Saccharomyces* strains produced wines with stronger fruity and floral flavor and a weaker herbal flavor.

### 3.4. Volatile Composition of Wines

A total of fifty-four volatile compounds were identified and quantified in wine samples produced by different yeast strains and fermentation protocols (Table 2).

However, in order to better assess the individual contribution of each quantified volatile compound to the overall wine aroma, odor activity value (OAV) and relative odor contribution (ROC) for compounds present in concentrations higher than their corresponding odor thresholds are shown in Table 3 [20]. Although the compounds with OAV value over 1 (OAV ≥ 1) are considered directly and individually responsible for the aroma profile of the wine, it is very important to detect the rest of the volatile compounds with lower OAV values, because they contribute to the complexity of wine aroma through a synergistic effect.

The majority of detected compounds are formed during alcoholic fermentation, so it is not surprising that their concentrations were significantly affected by yeast strains. As can be seen, quantitatively, the largest group of the volatile compounds in wine were higher alcohols, which represent about 78% (control wine sample) to more than 85% of all identified compounds (wine samples produced in both fermentation protocols with *H. uvarum* S-2 isolate). Such high average content of these compounds in wine samples is in agreement with the literature data, which indicated that higher alcohols commonly represent 80–90% of the aromatic constituents of wine [52]. Independently of the fermentation protocol, the total content of higher alcohols was significantly higher in wine samples produced by native yeast strains than in the control wine sample. Similar results were found for wine produced in pure and sequential fermentation with native *C. famata* WB-1 or *Metschnikowia pulcherrima* B-5 yeast isolates [20], *Issatchenkia terricola* SLY-4, and *Pichia kudriavzevii* F2-24 [47]. Although higher alcohols are recognized as the compounds that positively contribute to the aroma and complexity of wine (in concentrations below 300 mg/L), a negative influence on quality is observed in wines where their concentration exceeds 400 mg/L [53]. The most abundant higher alcohols in all wine samples were 1-pentanol and 3-methyl-1-butanol (isoamyl alcohol). While methyl-1-butanol was more dominant in control wines, the highest concentration of 1-pentanol was detected in wines produced in fermentations with native yeast strains. Since their concentration was below threshold concentration, the effect of these higher alcohol aromas on the quality of analyzed Tamjanika wine samples is indistinguishable. In addition, Muscat wines from Sardinia and Bornova were also characterized by the highest concentration of 3-methyl-1-butanol and phenylethanol [54], but their concentration in Tamjanika grapes was significantly higher. Such differences may arise from differences in climate, winemaking, or viticulture practices. Recently, a significantly higher content of higher alcohol was observed in the production of Chardonnay by the same *C. famata* WB-1 isolates [20], which also confirms the importance of grape variety and the initial composition of grape juice for the wine aroma profile. On the other hand, phenylethyl alcohol was present only in the Tamjanika wine sample fermented with *H. uvarum* S-2, in both fermentation protocols. Concentrations of phenylethyl alcohol in both samples were higher than its OAV, contributing with fine flower (rose, lilac) and honey notes. Previous reports also indicated that *H. uvarum* is characterized as a good producer of phenylethyl alcohol [13]. The ability to produce higher alcohols is a strain-dependent characteristic, although grape amino acid and fermentation conditions are, also, considered important for their occurrence and concentration in wine [53,55]. Furthermore, in addition to the ability to contribute and modulate the aroma and complexity of wine, higher alcohols are also essential for the production of esters [23]. Therefore, a good strategy to increase the wine complexity and ester production through fermentation is to find and use yeast strains capable of higher alcohols overproduction.

The fatty acid concentrations in all wine samples were below 4 mg/L, which is expected to contribute aroma complexity and enhance cheese and butter aroma notes [56]. Both native yeast strains produced octanoic acid in concentrations higher than in the control sample and above its ODT value, while oleic acid was present just in samples fermented by yeast isolate *H. uvarum* S-2 and commercial *S. cerevisiae* QA23. A higher level of oleic acid detected in wine samples fermented by commercial yeast compared to non-*Saccharomyces* yeast isolates was consistent with a previous study that characterized *S. cerevisiae* as a better producer of medium-chain fatty acids than non-*Saccharomyces* [50]. Additionally, these compounds are important for wine aroma formation as essential substrates for the production of fatty acid ethyl esters [3].

Esters are one of the most important aroma compounds in wines, typically responsible for the floral and fruity aroma. Their concentration in wine is strongly dependent on the yeast strains, fermentation, and storage conditions, as well as on the concentration of fatty acids, higher alcohols, and their precursors [55]. Referring to the analyzed Tamjanika wine samples, a total of 24 different esters were identified and quantified, while 11 of them were present in a concentration higher than their corresponding OAV. Based on the calculated ROC values, esters were responsible for a major part of the aroma profile, with the total ROC of esters from 55% for the control wine sample to almost 84% for the wine sample fermented by *H. uvarum* S-2. Generally, the concentration of esters was significantly higher in the samples produced by non-*Saccharomyces* yeasts, especially in the case of ethyl octanoate, ethyl decanoate, ethyl hexanoate, and phenylethylacetate that impart fruity and flowery odors to wine [20]. The total amount of esters (with the OAV over 1) in the control samples was twice lower than in both the samples fermented with *H. uvarum* S-2 and 2.5 times lower than in both samples produced by *C. famata* WB-1. Although each ester follows different patterns and their concentration is dependent on the yeast strain and fermentation protocol, generally higher content was present in samples produced in sequential fermentation protocol. Another researcher also reported higher contents of esters in wine samples produced in sequential fermentations for different non-*Saccharomyces* species [13,47]. Independently of the yeast strain and fermentation protocol, ethyl hexanoate expressed the highest contribution to the aroma of all analyzed Tamjanika wine samples (ROC up to 50%), followed by ethyl decanoate (ROC up to 15.95%), ethyl octanoate (ROC up to 5.53%), and phenylethylacetate (ROC lower than 5%). Compared to the control sample, the total ethyl ester content was significantly higher in wines fermented by native *C. famata* WB-1 (from 9 to 28% higher for pure and sequential inoculation) and *H. uvarum* S-2 (from 52 to 42% higher for pure and sequential inoculation) yeasts. Furthermore, in our experiments, the commercial yeast strain produces Tamjanika wine with the lowest content of higher alcohol acetates, while sequential inoculation of selected yeast isolates resulted in an increase in the production of these compounds known to contribute to fruity notes (mainly banana and apple odor) of wine. These results indicate that the *C. famata* WB-1 and *H. uvarum* S-2 yeasts were able to esterify present higher alcohols better than the used commercial strain, providing about 7- and 2.5-times higher alcohol acetates than in a control sample, respectively. Knowing that the enzymatic formation of ethyl and acetate esters required specific enzymes (acyl-CoA synthetase, alcohol acetyltransferase) [3], such results were evidence that the used native yeast strains were able to produce ester-synthesizing enzymes in higher content compared to the commercial *S. cerevisiae* QA23 strain. Therefore, the significant differences in the OAV and ROC for each compound confirmed the influence of the yeast strains on the wine’s aromatic profile and justified the need to find the best possible match of the yeast species and grape variety.

For the recognizability of the characteristic and unique aroma of the Muscat grape, such as Tamjanika grape variety, different terpenes play a fundamental role. Linalool and geraniol are recognized as predominant terpenic aroma compounds present in Muscat grapes, along with citral, citronellol, nerol, and α-terpineol [57,58]. In accordance with the literature data, seven terpenes, p-cymene, γ-terpinene, terpinolene, linalool, (Z) and (E)-β-ocimene, and one C13-norisoprenoid (β-ionone), were present above the ODT and contribute to the overall aroma of analyzed Tamjanika wine samples (Table 2). The average total concentration of terpenes ranged between 0.25 and 0.98 mg/L, for control and pure fermentation with *C. famata* WB-1, while only p-cymene and terpinolene were present in all samples. The concentration of individual terpenes quantified in analyzed Tamjanika wine samples was similar to the levels of p-cymene (5–35 µg/L), γ-terpinene (6–35 µg/L), nerol (3–43 µg/L), and linalool (6–375 µg/L) averagely present in different Muscat wines [59]. According to their ROC values of up to 12%, these compounds could significantly affect the wine aroma and sensory profile of Tamjanika wines regardless of their low concentration in the samples. Linalool, which positively contributes to the floral notes, was presented with OAV > 1 (from 3.2 to 4.8 for sequential and pure fermentation, respectively) in wines fermented by *H. uvarum* S-2, but was not detected in wines fermented by *C. famata* WB-1 and commercial *S. cerevisiae* QA23 strain. In addition, compared to the pure fermentations (independently of used strains), sequential fermentation elicited higher concentrations of p-cymene, terpinolene, β-ionone, and (Z) and (E)-β-ocimene. Similar results, where different non-*Saccharomyces* strains positively affected the transformation and release of varietal aromas, were previously reported for some aromatic grape varieties such as Muscat, Gewurztraminer, Sauvignon blanc, and Verdejo [9,60,61]. Although the total concentration of all terpenes was higher for wines fermented by native strains (almost 1 mg/L and 0.9 mg/L for *C. famata* WB-1 and *H. uvarum* S-2, respectively) than for *S. cerevisiae* QA23 fermentation alone (0.253 mg/L), the total ROC of terpenes for the control sample was the highest. This finding can be explained by the lowest number of quantified key compounds with OAV above 1 in the control sample.

Furthermore, the release and transformation of varietal compounds, which are specific for certain grape varieties and are mainly present in odorless form as aroma precursors [60,61], is possible naturally during winemaking, but as a very slow process. Hence, finding and using yeast strains with enhanced glucosidase enzyme activity should be considered a good strategy for increasing the terpenoid aromas in wines.

**Figure 3:**
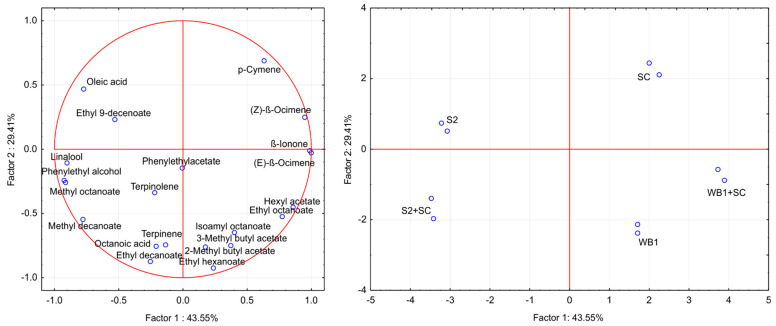
Principal component analysis (PCA) of volatile compounds (OAV > 1) from Tamjanika wine samples fermented in pure or sequential inoculation with *C. famata* WB-1, *H. uvarum* S-2, and *S. cerevisiae* QA23 (SC). Data were the mean of two biological replicates of fermentation.

The PCA was used to visualize and reveal the diversity or common characteristics among the wine samples produced by different yeast strains (Figure 3).

According to the PCA, four factors (principal components) had eigenvalues higher than 1, cumulatively explaining 99% of total variance of the initial data set. PC1 and PC2 comprised 74.52% of the variability. A clear differentiation among the Tamjanika wine samples can be observed, while it was found that samples fermented by the same yeast isolates were relatively similar because they were loaded closer to each other in the PCA plot. Methyl decanoate, methyl octanoate, phenylethyl alcohol, and linalool showed high negative loading scores in PC1 that distinguished both wine samples fermented by *H. uvarum* S-2 from the other samples. However, samples fermented by *H. uvarum* S-2 were negatively correlated with p-cymene and β-ocimene. On the other hand, Tamjanika wines produced in both inoculation protocols by *C. famata* WB-1 were located in the lower right quadrant (positive values for PC1 and the negative for PC2). The wine produced in pure fermentation with *C. famata* WB-1 was characterized by a higher relative contribution of acetate esters, isoamyl octanoate, and ethyl hexanoate. These compounds were related to fruit, herb, and pleasant aroma notes. The control wine sample was clearly different from the wine samples fermented by both native yeast strains in both inoculation protocols, with p-cymene as the compound that was considered most responsible for wine aroma.

## 4. Conclusions

This study indicates that both *C. famata* WB-1 and *H. uvarum* S-2 strains can be considered as alternative starter cultures, since they are able to positively modulate wine quality parameters, along with the aroma and sensory profile of the wine, in both pure and sequential fermentation on the industrial level. We have shown that native non-*Saccharomyces* strains can serve as a tool for ensuring the unique aroma profiles, while the sensory analysis demonstrated that all Tamjanika wines produced in different fermentations resulted in a similar taste profile, but with statistically different scores for the majority of gustatory attributes.

The fact that native yeast strains proved good fermentation capability, and that a variety of aroma compounds were produced in different Tamjanika wine samples, proves the uniqueness of the sensory and aroma profile of yeast isolates–grape combination, indicating its potential in the winemaking industry.

## Figures and Tables

**Table 1:** Standard quality parameters of Tamjanika wine samples produced in pure and sequential fermentations inoculated with *C. famata* WB-1, *H. uvarum* S-2, and *S. cerevisiae* QA23 (control).

Parameter	Pure Fermentation			Sequential Fermentation	
	*Candida* *famata* WB-1	*Hanseniaspora uvarum* S-2	*Saccharomyces cerevisiae* QA23	*Candida* *famata* WB-1	*Hanseniaspora uvarum* S-2
Ethanol, % *v*/*v*	12.79 ± 0.30 a	13.05 ± 0.09 a	13.85 ± 0.10 b	13.32 ± 0.17 c	13.49 ± 0.20 c
Total extract, g/L	21.2 ± 0.50 a	24.5 ± 0.80 b	18.5 ± 0.58 c	19.60 ± 0.90 c	21.4 ± 0.58 a
Total acids (as tartaric acid), g/L	6.35 ± 0.13 a	5.96 ± 0.32 ad	4.92 ± 0.13 b	4.39 ± 0.27 c	5.62 ± 0.13 ad
Volatile acids (as acetic acid), g/L	0.60 ± 0.06 a	0.59 ± 0.05 a	0.42 ± 0.03 b	0.62 ± 0.04 a	0.48 ± 0.02 c
Reducing sugar g/L	2.03 ± 0.08 a	3.81 ± 0.31 b	2.38 ± 0.18 c	1.35 ± 0.10 d	1.79 ± 0.18 e
Free SO_2_, mg/L	37 ± 2.10 a	69 ± 1.90 b	12 ± 1.10 c	35 ± 1.80 ad	33 ± 1.40 ad
Total SO_2_, mg/L	87 ± 2.80 a	105 ± 4.20 b	71 ± 2.30 c	98 ± 2.80 d	102 ± 3.50 d
pH	3.19 ± 0.02 a	3.31 ± 0.05 b	3.65 ± 0.04 c	3.69 ± 0.09 c	3.62 ± 0.11 c

Data were the mean ± SD of two biological replicates of fermentation. Different letters in the same row show significant differences according to the analysis of variance at *p* < 0.05 (Tukey’s HSD test).

**Table 2:** Concentration of volatile components (in mg/L) found in the Tamjanika wine samples produced in pure and sequential fermentations inoculated with *C. famata* WB-1, *H. uvarum* S-2, and *S. cerevisiae* QA23 (control).

Parameter	Aroma Descriptor	ODT, mg/L	Pure Fermentation			Sequential Fermentation	
			*Candida* *famata* WB-1	*Hanseniaspora uvarum* S-2	*Saccharomyces cerevisiae* QA23	*Candida* *famata* WB-1	*Hanseniaspora uvarum* S-2
**Higher alcohols**							
Isobutanol	Wine, solvent, bitter	40 *	nd	nd	0.11 ± 0.00	nd	nd
3-Methyl-1-butanol	Whiskey, malt, burnt	30 *	10.72 ± 0.35 a	10.23 ± 0.14 a	16.51 ± 1.21 b	11.86 ± 0.23 c	9.52 ± 0.31 d
2-Methyl-1-butanol	Malt	30 *	3.83 ± 0.01 a	4.14 ± 0.09 b	tr	4.07 ± 0.02 b	4.57 ± 0.03 c
1-Pentanol	Bitter, almond, balsamic	64 *	30.14 ± 0.63 a	38.09 ± 1.31 b	21.31 ± 0.64 c	47.74 ± 1.25 d	35.91 ± 0.92 e
4-Methyl-1-pentanol	Almond, toasted	50 *	nd	nd	0.01 ± 0.00	nd	nd
2,3-Butanediol	Butter, creamy	668 *	7.73 ± 0.01 a	nd	nd	8.81 ± 0.08 b	nd
Phenylethyl alcohol	honey, spice, rose, lilac	14 *	tr	16.26 ± 0.60 a	tr	tr	22.52 ± 0.71 b
Total higher alcohols			52.42 ± 1.01	68.72 ± 2.14	37.94 ±1.85	72.48 ± 1.58	72.52 ± 1.97
**Acids**							
Hexanoic acid	Cheese, oily	0.42 **	tr	0.02 ± 0.00 a	0.02 ± 0.00 a	0.018 ± 0.02 a	tr
Octanoic acid	Sweet, cheese	0.5 **	2.97 ± 0.00 a	1.50 ± 0.09 b	0.44 ± 0.00 c	0.90 ± 0.01 d	1.77 ± 0.04 e
Decanoic acid	Rancid, fat	1 **	tr	0.01 ± 0.00	nd	nd	tr
Oleic acid	Fat	0.5 *	nd	1.33 ± 0.02 a	2.28 ± 0.12 b	nd	2.19 ± 0.09 b
Total acids			2.97 ± 0.00	2.86 ± 0.09	2.74 ± 0.12	0.92 ± 0.03	1.77 ± 0.13
**Esters**							
Ethyl lactate	Fruit, butter	154 *	nd	nd	0.01 ± 0.00	nd	nd
Isopentyl acetate	Banana	30 *	nd	nd	1.72 ± 0.17	nd	nd
Ethyl hexanoate	Apple peel, fruit	0.014 *	0.79 ± 0.00 a	0.47 ± 0.04 b	0.17 ± 0.00 c	0.88 ± 0.04 d	0.80 ± 0.02 a
Ethyl butanoate	Pineapple, apple, peach	20 *	tr	nd	0.01 ± 0.00	tr	tr
3-Methyl butyl acetate	Banana	0.03 *	0.04 ± 0.00 a	0.03 ± 0.00 b	Nd	0.06 ± 0.00 c	0.04 ± 0.00 a
2-Methyl butyl acetate	Fruity, fatty, pleasant	0.05 **	0.83 ± 0.04 a	0.36 ± 0.03 b	Nd	0.33 ± 0.01 b	0.22 ± 0.01 c
Hexyl acetate	Fruit, herb	0.67 *	2.78 ± 0.12 a	tr	Tr	2.41 ± 0.04 b	tr
Ethyl levulinate	-		nd	0.01 ± 0.00 a	Nd	nd	0.01 ± 0.00 a
Ethyl 3-furoate	-		nd	nd	0.05 ± 0.00	nd	nd
Methyl heptanoate	-		nd	nd	nd	nd	0.01 ± 0.00
Methyl octanoate	Orange	0.2 *	tr	0.64 ± 0.01 a	tr	tr	0.99 ± 0.04 b
Diethyl succinate	Wine, fruit	200 *	tr	0.72 ± 0.05 a	0.87 ± 0.04 b	0.83 ± 0.05 b	0.77 ± 0.02 a
Ethyl octanoate	Fruit, fat	0.58 *	2.25 ± 0.12 a	1.52 ± 0.09 b	1.43 ± 0.02 b	2.77 ± 0.11 c	1.96 ± 0.06 d
Isoamyl hexanoate	-		0.60 ± 0.01 a	0.40 ± 0.00 b	tr	tr	tr
Phenylethylacetate	Rose, honey, tobacco	0.25 **	0.97 ± 0.04 a	0.87 ± 0.02 b	0.55 ± 0.00 c	0.45 ± 0.01 d	0.35 ± 0.00 e
Ethyl nonanoate			tr	0.48 ± 0.02	nd	nd	tr
Methyl decanoate	Wine	0.05 †	0.05 ± 0.00 a	tr	tr	tr	0.11 ± 0.00 b
Ethyl 9-decenoate	Green, fruity, fatty	0.1 **	tr	0.24 ± 0.01 a	0.05 ± 0.00 b	0.06 ± 0.01 b	0.05 ± 0.00 b
Ethyl decanoate	Grape	0.2 **	1.79 ± 0.06 a	1.83 ± 0.03 ab	1.46 ± 0.11 c	1.82 ± 0.10 ab	1.89 ± 0.03 b
Isoamyl octanoate	-	0.125 **	0.12 ± 0.01 a	0.09 ± 0.00 b	0.06 ± 0.00 c	0.17 ± 0.01 d	0.14 ± 0.01 a
Ethyl dodecanoate	Sweet, floral, soapy	0.8 **	0.44 ± 0.02 a	0.38 ± 0.02 b	0.08 ± 0.00 c	0.17 ± 0.00 d	0.35 ± 0.02 b
Ethyl myristate	Sweet fruity, fatty, butter	2 **	tr	tr	0.01 ± 0.00	tr	tr
Ethyl palmitate	Wax, fatty	2 **	tr	0.07 ± 0.00 a	tr	tr	0.09 ± 0.00 b
Ethyl 9-octadecanoate	-		nd	2.16 ± 0.09 a	nd	0.91 ± 0.01 b	tr
Total esters			10.61 ± 0.42	12.00 ± 0.41	4.74 ± 0.34	10.86 ± 0.39	7.78 ± 0.61
**Terpenes**							
α-Terpinene	Lemon	0.25 ‡	tr	0.01 ± 0.00	tr	tr	tr
p-Cymene	Solvent, minty, citrus	0.011 ‡	0.034 ± 0.00 a	0.043 ± 0.01 b	0.06 ± 0.00 c	0.06 ± 0.00 c	0.02 ± 0.00 d
Limonene	Lemon, orange	0.015 **	tr	0.01 ± 0.00	tr	tr	nd
(Z)-β-Ocimene	Citrus, herb, flower	0.034 #	0.055 ± 0.00 a	tr	0.083 ± 0.00 b	0.12 ± 0.01 c	tr
(E)-β-Ocimene	Sweet, herb	0.034 #	0.11 ± 0.01 a	tr	0.072 ± 0.00 b	0.167 ± 0.03 c	tr
p-Mentha-3,8-diene	-		tr		tr	0.07 ± 0.00	
γ-Terpinene	Woody, citrus	0.26 ¥	0.75 ± 0.03 a	0.71 ± 0.02 a	nd	0.45 ± 0.02 b	0.48 ± 0.01 b
Terpinolene	Piney	0.041 ¥	0.036 ± 0.00 a	0.012 ± 0.00 b	0.038 ± 0.00 a	0.046 ± 0.00 c	0.094 ± 0.00 d
Linalool	Flower, lavender	0.025 *	tr	0.12 ± 0.00 a	nd	tr	0.08 ± 0.00 b
1,3,8-p-Menthatriene	Turpentine		tr	nd	nd	0.70 ± 0.04	nd
allo-Ocimene	-		0.042 ± 0.00 a	nd	0.047 ± 0.00 b	0.167 ± 0.02 c	tr
Nerol	Rose, flower	0.3 *	tr	nd	0.01 ± 0.00	tr	nd
Citronellol	Sweet, citrus-like	0.10 **	tr	nd	nd	0.01 ± 0.00	nd
Total terpenes			1.027 ± 0.04	0.905 ± 0.03	0.310 ± 0.00	1.79 ± 0.12	0.674 ± 0.01
**C13-norisoprenoid**							
β-Ionone	Balsamic, rose, violet	7 × 10^−6^ ¥	0.0004 ± 0.00 a	tr	0.0003 ± 0.00 b	0.0008 ± 0.00 c	tr
**Other**							
Dimethyl sulfate	-				0.536 ± 0.14		
Diethyl sulfate			nd	nd	0.01 ± 0.00	tr	nd
Benzaldehyde	Almond	0.35 *	0.07 ± 0.00 a	0.05 ± 0.00 b	0.15 ± 0.01 c	0.07 ± 0.00 a	0.06 ± 0.00 d
Levoglucosenone	-		nd	0.01 ± 0.00	tr	nd	tr
4-Vinylguaiacol	Spices, curry	0.04 ‡	0.01 ± 0.00	nd	nd	nd	nd

ODT—Odor detection threshold; nd—not detected; tr—could not be quantified (trace). Data were the mean ± SD of two biological replicates of fermentation. Different letters in the same row show significant differences according to the analysis of variance at *p* < 0.05 (Tukey’s HSD test). Aroma descriptor and odor detection threshold values are taking from * [46], ** [47], ‡ [48], † [49], # [50], ¥ [51].

**Table 3:** Odor activity values (OAV) and relative odor contribution (ROC) of key aroma compounds in Tamjanika wine samples.

Compound	Pure Fermentation						Sequential Fermentation			
	*Candida famata* WB-1		*Hanseniaspora* *uvarum* S-2		*Saccharomyces* *cerevisiae* QA23		*Candida famata* WB-1		*Hanseniaspora* *uvarum* S-2	
	OAV	ROC, %	OAV	ROC, %	OAV	ROC, %	OAV	ROC, %	OAV	ROC, %
**Higher alcohol**										
Phenylethyl alcohol	0.00	0.00	1.16	1.40	0.00	0.00	0.00	0.00	1.61	1.59
**Acids**										
Octanoic acid	5.94	4.97	3.00	3.62	0.88	1.92	1.80	1.47	3.54	3.49
Oleic acid	0.00	0.00	2.66	3.21	4.56	9.97	0.00	0.00	4.38	4.32
**Esters**										
Ethyl hexanoate	56.43	47.20	33.57	40.49	12.14	26.54	62.86	51.24	57.14	56.35
3-Methyl butyl acetate	1.33	1.12	1.00	1.21	0.00	0.00	2.00	1.63	1.33	1.31
2-Methyl butyl acetate	16.60	13.89	7.20	8.68	0.00	0.00	6.60	5.38	2.40	2.37
Hexyl acetate	4.15	3.47	0.00	0.00	0.00	0.00	3.60	2.93	0.00	0.00
Methyl octanoate	0.00	0.00	3.20	3.86	0.00	0.00	0.00	0.00	4.95	4.88
Ethyl octanoate	3.88	3.25	2.62	3.16	2.47	5.39	4.78	3.89	3.38	3.33
Phenylethylacetate	3.88	3.25	3.48	4.20	2.20	4.81	1.80	1.47	0.60	0.59
Methyl decanoate	0.00	0.00	1.02	1.23	0.00	0.00	0.00	0.00	2.20	2.17
Ethyl 9-decenoate	0.00	0.00	2.40	2.89	0.50	1.09	0.60	0.49	0.50	0.49
Ethyl decanoate	8.95	7.49	9.15	11.04	7.30	15.95	9.10	7.42	9.10	8.97
Isoamyl octanoate	0.96	0.80	0.72	0.87	0.48	1.05	1.36	1.11	1.12	1.10
**Terpenes**										
p-Cymene	3.09	2.59	3.91	4.71	5.45	11.92	5.45	4.45	1.82	1.79
(Z)-β-Ocimene	1.62	1.35	0.00	0.00	2.44	5.34	3.53	2.88	0.00	0.00
(E)-β-Ocimene	3.24	2.71	0.00	0.00	2.12	4.63	4.91	4.00	0.00	0.00
γ-Terpinene	2.88	2.41	2.73	3.29	0.00	0.00	1.73	1.41	1.85	1.82
Terpinolene	0.88	0.73	0.29	0.35	0.93	2.03	1.12	0.91	2.29	2.26
Linalool	0.00	0.00	4.80	5.79	0.00	0.00	0.00	0.00	3.20	3.16
**C13-norisoprenoid**										
β-Ionone	5.71	4.78	0.00	0.00	4.29	9.37	11.43	9.32	0.00	0.00

OAV—ratio between the mean concentration of certain aroma compound in wine sample and its ODT value. ROC—ratio between the OAV of the individual compound and the total OAV of each wine sample.

## Data Availability

The data presented in this study are available on request from the corresponding author.
